# New insights in the natural course of eosinophilic esophagitis

**DOI:** 10.1002/ueg2.12702

**Published:** 2024-11-07

**Authors:** Gwen M. C. Masclee, Albert Jan Bredenoord

**Affiliations:** ^1^ Department of Gastroenterology and Hepatology Amsterdam University Medical Center Amsterdam The Netherlands

**Keywords:** cancer, EoE, epidemiology, fibrosis, gender, histological remission, pathophysiology, sex, therapy, treatment

A tremendous amount of knowledge has been added on eosinophilic esophagitis (EoE) in the past decade: insights in pathophysiological mechanisms have resulted in new therapeutic drug targets.^1^ Insights on the natural course of EoE made it clear that histological remission may be required to prevent fibrosis formation in the esophagus.^2^

In the current UEG journal two articles provide new insights into the epidemiology of EoE: sex‐related differences in EoE and long‐term consequences of inflammation and the association with cancer.

The article by Laserna‐Mendieta et al.^3^ reports on sex‐related differences in EoE phenotype and treatment choices for patients with EoE. Prospectively collected data from the EoE CONNECT study (38 medical centers located in 4 European countries) allowed a cross‐sectional analysis among 2976 EoE patients showing that males more often have a fibrotic phenotype whereas female EoE patients tend to experience more symptoms. A possible explanation for the observation of more fibrosis in male EoE patients may be a longer time before diagnosis and treatment onset as they are diagnosed at a later age. However, as the authors report: diagnostic delay and number of previous gastroscopies was similar between male and female patients. This raises the question regarding potential sex‐related differences in pathophysiology, symptom presentation, treatment and disease progression. Regarding the latter issue, it is postulated that estrogens may have a protective role in fibrogenesis.^4^ Therapeutic approach (proton pump inhibitor, swallowed topical steroids) and efficacy in terms of clinical and histological remission did not differ between males and females in the current study by Laserna‐Mandieta et al. Then, what can we learn from other diseases? Much attention has been given lately to symptom presentation differences between males and females in acute cardiovascular conditions, leading to later recognition of acute cardiovascular issues and worse outcomes in females.^5^ Taking this in consideration, as clinicians we should acknowledge the facts that fibrosis may occur more often and sooner in male patients—and therapy with dilation should be employed. On the other hand we should realize that female patients tend to have a higher symptom burden—and thus therapeutic strategies to reduce inflammation and functional symptoms should be the optimal therapeutic target for females.

From several diseases such as inflammatory bowel disease, primary sclerosing cholangitis, lichen and reflux disease we have learned that chronic inflammation leads to a tissue damage‐repair cycle which may increase the risk of malignancy. In the article by Uea^6^ it was investigated whether EoE is associated with an increased risk of cancer. They did so by employing a nationwide population‐based cohort study using pathology registers in Sweden including in total 1580 EoE patients and matching these to the general population and to siblings. Overall, only two EoE patients of the entire cohort were diagnosed with esophageal cancer, and in total 45 other types of cancers were identified during a relatively short median follow‐up of 7 years. One should realize that although the calculated risk of cancer may be increased in the study, the absolute number of patients diagnosed with esophageal cancer is rather low. Secondly, generalizability may be difficult as the control group consisted of the general population or siblings, which does not rule out the influence of health seeking behavior and ‘enhanced’ surveillance among EoE patients under secondary or tertiary care with increased likelihoods of receiving stricter follow‐up compared to the general population, although almost half of the EoE patients did not have a follow‐up endoscopy during follow‐up. Nevertheless, to date this is the largest EoE study assessing epidemiological associations between diagnosed EoE and malignancy showing that although the number of cancer cases were small, the risk of (esophageal) cancer in EoE may not be negligible. This study^6^ highlights our future need to investigate the influence of ongoing histological inflammation and subsequent risk of dysplasia and malignancy—as we have previously learned from the cascade of reflux esophagitis, Barrett's esophagus and esophageal adenocarcinoma.^7^ In order to answer this research question we should take the following issues into account: (1) accurate and valid data is needed, (2) given the often relatively young age at EoE diagnosis, patients should have a (life) long‐follow up to develop malignancy, (3) assessing associations between disease (exposure) and cancer (outcome) is often difficult given the low incidence of the outcome. To which extent effective EoE therapy may influence and hopefully decrease the risk of malignancy is the next step to consider.

## CONFLICT OF INTEREST STATEMENT

AJB received research funding from Nutricia, Thelial, Sanofi/Regeneron, SST, and Dr. Falk Pharma and received speaker and/or consulting fees from Laborie, Medtronic, BMS, Dr. Falk Pharma, Calypso Biotech, Eupraxia, Aqilion, Alimentiv, Sanofi/Regeneron, Reckitt and AstraZeneca.

## Data Availability

Data sharing is not applicable to this article as no new data were created or analyzed in this study.
